# Supplementary material for: Supplementation with a New Standardized Extract of Green and Black Tea Exerts Antiadipogenic Effects and Prevents Insulin Resistance in Mice with Metabolic Syndrome
Source: Int J Mol Sci. 2023 May 10;24(10):8521. doi: 10.3390/ijms24108521 (PMC10218622; doi:10.3390/ijms24108521)
Supplement: Supplementary file 1 [file ijms-24-08521-s001.zip › ijms-2340748-supplementary.pdf]

SUPPLEMENTARY INFORMATION

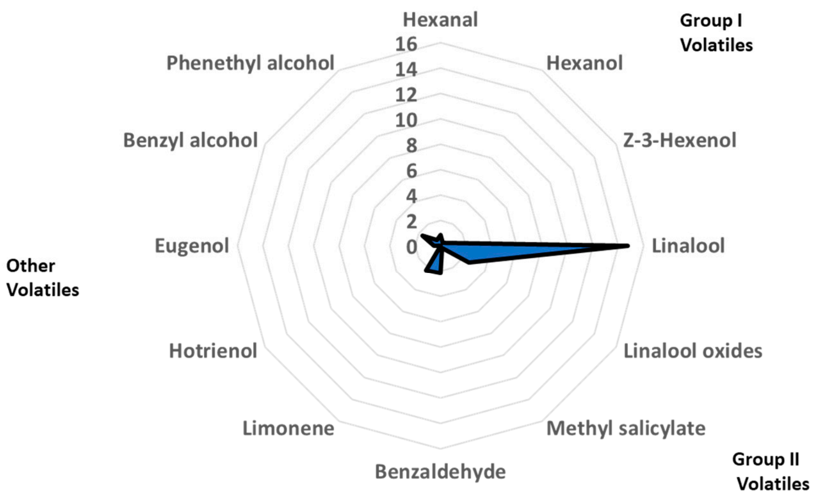

.

Figure S1. Volatile composition (relative proportion, %) of Complex Tea Extract analyzed by gas chromatography.

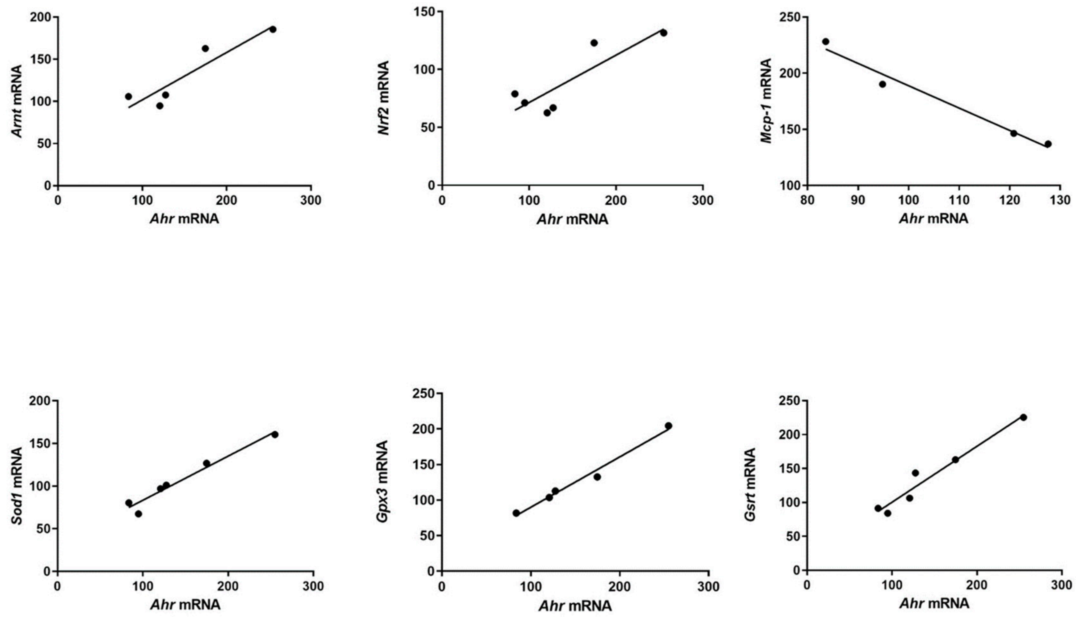

Figure S2. Correlation between AhR gene expression and antioxidants, pro-inflammatory and AHR-pathway genes in gastrocnemius tissue in mice supplemented with green–black tea extract. Values are represented as mean ± SEM; n=6-8 mice/group.
